# Complex left atrial appendage closure using 3D printing system simulation in a patient with mitral prosthetic valve: a case report

**DOI:** 10.1093/ehjcr/ytae574

**Published:** 2024-10-23

**Authors:** Luca Vicini Scajola, Antonio Sanzo, Giulia Magrini, Stefania Marconi, Roberto Rordorf

**Affiliations:** Department of Molecular Medicine, School of Cardiology, University of Pavia, Via Adolfo Ferrata 5, 27100 Pavia, Italy; Arrhythmia and Electrophysiology Unit, IRCCS Fondazione Policlinico S. Matteo, Viale Camillo Golgi 19, 27100 Pavia, Italy; Division of Cardiology, Fondazione IRCCS Policlinico San Matteo, Viale Camillo Golgi 19, 27100 Pavia, Italy; Arrhythmia and Electrophysiology Unit, IRCCS Fondazione Policlinico S. Matteo, Viale Camillo Golgi 19, 27100 Pavia, Italy; Division of Cardiology, Fondazione IRCCS Policlinico San Matteo, Viale Camillo Golgi 19, 27100 Pavia, Italy; Division of Cardiology, Fondazione IRCCS Policlinico San Matteo, Viale Camillo Golgi 19, 27100 Pavia, Italy; Department of Civil Engineering and Architecture, University of Pavia, Viale Adolfo Ferrata 5, 27100 Pavia, Italy; Arrhythmia and Electrophysiology Unit, IRCCS Fondazione Policlinico S. Matteo, Viale Camillo Golgi 19, 27100 Pavia, Italy; Division of Cardiology, Fondazione IRCCS Policlinico San Matteo, Viale Camillo Golgi 19, 27100 Pavia, Italy

**Keywords:** Atrial fibrillation, Left atrial appendage closure, 3D printing, Case report

## Abstract

**Background:**

Left atrial appendage (LAA) closure (LAAc) has emerged as a safe and effective alternative to oral anticoagulation (OAC) for stroke prevention in patients with atrial fibrillation (AF) and contraindications to OAC.

**Case summary:**

A 61-year-old woman with permanent AF and a history of mitral surgery replacement with mechanical prosthesis was referred to our cardiology department to undergo LAAc. The preoperative computed tomography (CT) revealed that the ostium of the LAA was close to the mitral prosthesis ring. As a result of the difficult LAA morphology, a CT image-based virtual model was created, then a 3D printing model was prepared in our laboratory, and procedure simulation was performed with the two different LAA occlusion devices (plug- vs. pacifier-like models) to see which one was more suitable for the patient anatomy.

**Discussion:**

In this case of complex LAAc in a patient with mechanical prosthetic mitral valve, the use of a 3D printed model has guided prosthesis selection and device sizing reducing procedure time and complications.

**Learning points**

- 3D printed model technology and simulation can help in complex cases reducing procedure time and complications.

## Introduction

Stroke event prevention with anticoagulant therapy is indicated in patients with atrial fibrillation (AF) and high ischaemic risk^1^ (CHADS2-VASC score ≥ 2). However, in some cases, there may be contraindications to oral anticoagulation (OAC) or even the presence of persistent thrombosis despite adequate therapy. Furthermore, the presence of recurrent cerebral ischaemic events despite adequate anticoagulant therapy [malignant left atrial appendage (LAA)] has been proposed as an alternative indication for LAA closure (LAAc) procedure.^1^ Left atrial appendage closure has emerged as a safe and effective alternative to OAC for stroke prevention in patients with AF.^2^

## Summary figure

**Figure:**
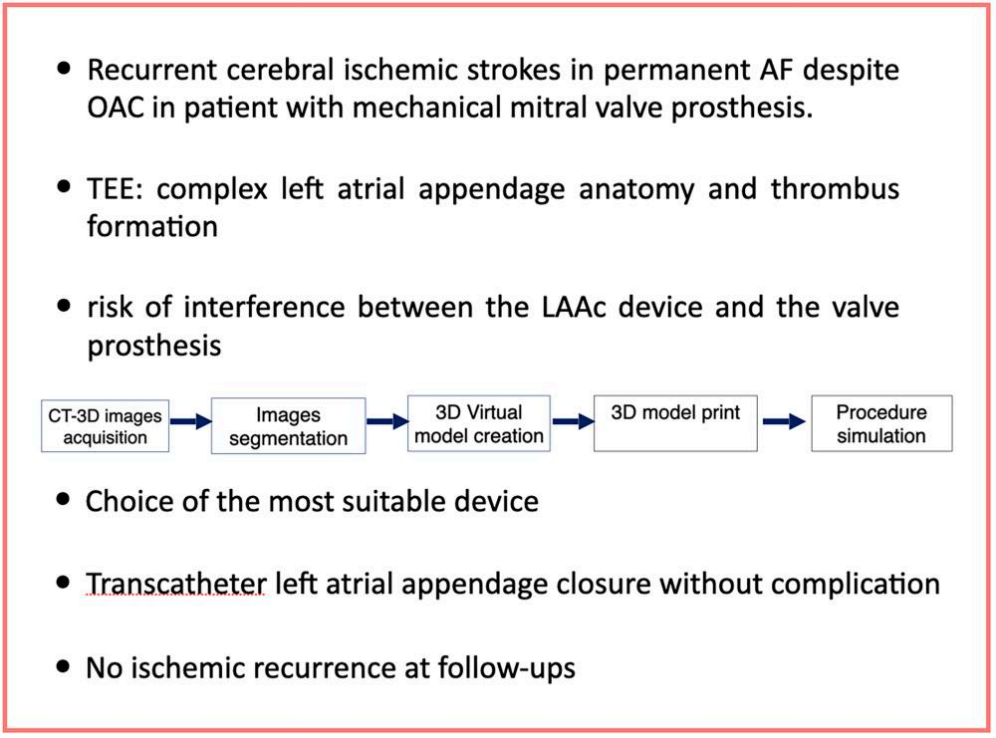


## Case presentation

A 61-year-old woman with permanent AF was referred to our cardiology department for LAAc after a transient cerebral ischaemic episode in September 2021 followed by two ischaemic strokes in November 2021 and January 2022 despite adequate anticoagulant therapy with INR value in therapeutic range. On June 2021, she underwent mitral surgery replacement with a bileaflet mechanical prosthesis. Her CHA_2_DS_2_-VASc score was 4 and HAS-BLED score was 2. Her current medical therapy included OAC with vitamin K antagonist in addition to antihypertensive drugs and beta-blockers. On March 2023, a transoesophageal echocardiography study showed a LAA spontaneous echocontrast and thrombosis in formation. On physical examination, the patient was afebrile, asymptomatic for dyspnoea, with a normal breathing rate. The pulse rate was arrhythmic at 77 b.p.m. Only prosthetic sounds were detected upon cardiopulmonary auscultation. The abdomen was non-tender to palpation, and there was no peripheral oedema. Electrocardiogram at admission showed AF with normal ventricular rate; no relevant alterations were seen in blood chemistry tests. Before the procedure, warfarin dose was reduced in order to obtain an INR value around 2. The patient underwent transoesophageal echocardiography that showed multilobed LAA with spontaneous echocontrast but no thrombosis, non-dilated left ventricle with preserved function, normally functioning mitral prosthesis, and left atrial severe dilation. The preoperative computed tomography (CT) revealed that the ostium of the LAA was close to the mitral prosthesis ring.

**Figure 1:**
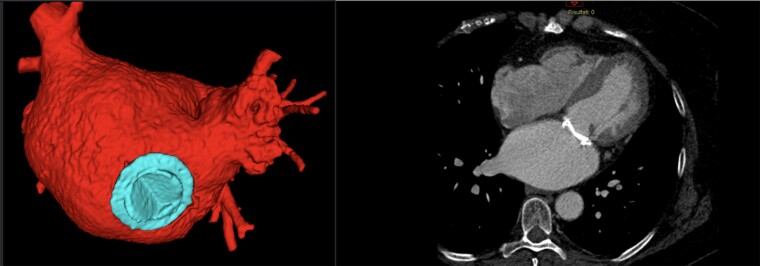
Computed tomography–based three-dimensional virtual model segmentation and computed tomography scan.

In consideration of the resolution of the thrombotic formation inside the LAA, the use of intraprocedural cerebral protection was considered unnecessary. As a result of the difficult LAA morphology, the proximity of the ostium to the prosthetic valve ring, and the difficult access to the inter-atrial septum due to previous transseptal cardiac surgical approach, a CT image-based virtual model was created (*Figure 1*). Then, a 3D printing model was prepared in our laboratory, and procedure simulation was performed from inferior and superior access point with the two different LAA occlusion devices (plug- vs. pacifier-like models) to see which one was more suitable for the patient anatomy (see Supplementary Material online, Video S1). For the production of the 3D printed model, we utilized material jetting technology, a high-resolution method capable of combining multiple photopolymers at the microscale level. Like all 3D printing technologies, the machine deploys the material in a layer-by-layer fashion. The 3D printed model was produced using a proprietary soft material, Agilus 30 Clear, a 30 Shore A photopolymer, to print the atrial wall. Additionally, a mixture of Agilus 30 Clear and a rigid photopolymer, VeroMagenta, was used to print the prosthetic valve. The resulting mixture features a Shore A hardness value of 95.

**Figure 2:**
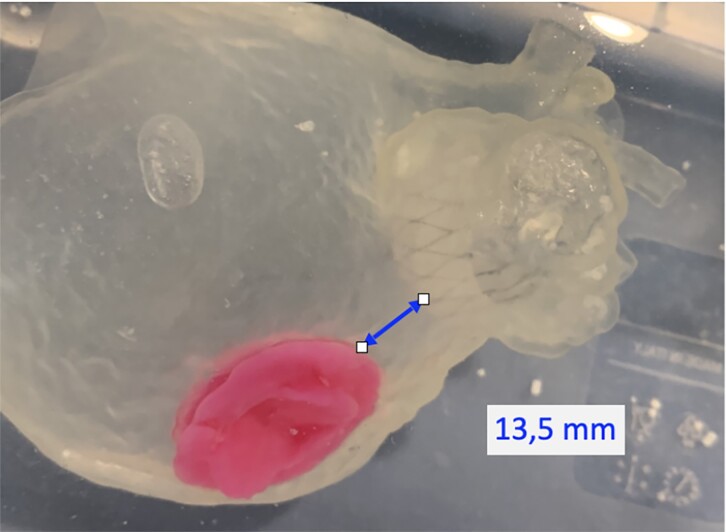
Three-dimensional printed model with plug device.

**Figure 3:**
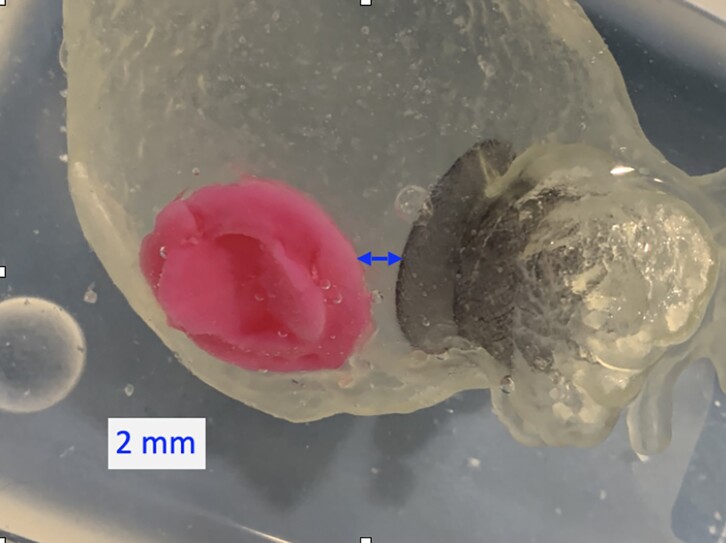
Three-dimensional printed model with pacifier principle device.

As can be seen from the images of the 3D printed reconstruction (*Figures 2* and *3*), the plug-type device allowed a greater safety margin from the mechanical valve prosthesis compared to the pacifier-like model (13.5 mm vs. 2 mm), thus guaranteeing a more straightful approach to the LAA.

At intervention time, transseptal puncture at an inferior–posterior site of fossa ovalis^3^ using a right femoral approach was performed under transesophageal echocardiogram (TEE) guidance and plug LAA occlusion device was successfully implanted. Based on previously acquired 3D printing model and LAA measures performed both with TEE and LAA angiography, a 35 mm models was chosen. Three-dimensional reconstruction with ecoTOE was used during the first evaluation and allowed to better address the complexity of the case. It was also used during the procedure to verify the correct positioning of the device after LA closure. No complications occurred during the procedure.

**Figure 4:**
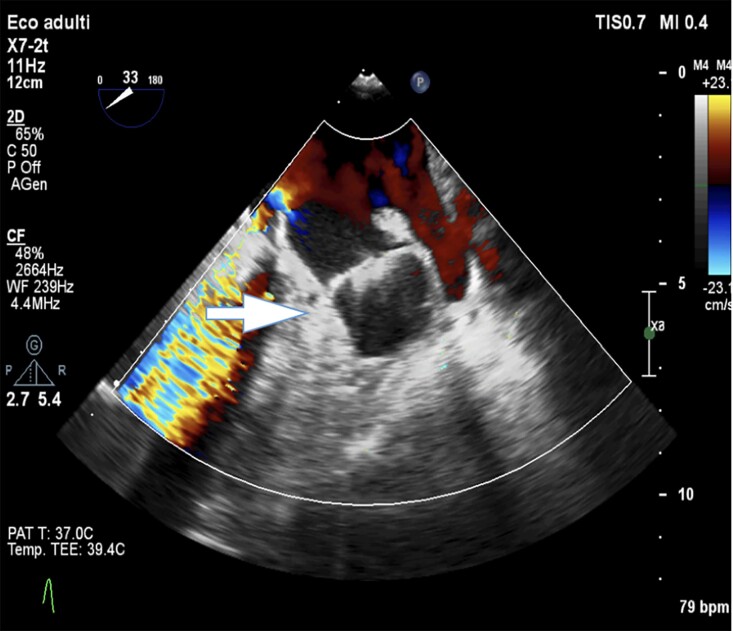
One-month follow-up with transesophageal echocardiogram.

At 1-month follow-up visit, transoesophageal echocardiography imaging demonstrated complete LAA occlusion and correct placement of the device with an adequate compression and no residual leak (*Figure 4*). At 6-month follow-up, the patient is asymptomatic with no further cerebral ischaemic episodes on warfarin therapy.

## Conclusions

Left atrial appendage closure has been suggested as an alternative option for stroke prevention in patients with AF who are unsuited to anticoagulation therapy, as stated in the most recent European Society of Cardiology guidelines. As this technique is now increasingly and widely used, more challenging cases will be faced up with the need for additional tools to better understand and overcome critical issues. In this case of complex LAAc in a patient with mechanical prosthetic mitral valve, studying the patient’s anatomy with a 3D printed model has guided prosthesis selection and device sizing potentially reducing procedure time and complications as previously described.^4^ Moreover, it also allowed operators to perform a step-by-step simulation of the procedure, allowing them to simulate movements with the materials and have a direct contact feedback. The physical model can be also produced using appropriate techniques and materials to closely resemble the mechanical properties of real tissue, allowing for the testing of interventions on patient-specific geometry.

## Supplementary Material

ytae574_Supplementary_Data

## Data Availability

The data underlying this article are available in the article and in its online supplementary material.

## Lead author biography

Luca Vicini Scajola is a cardiology resident at the Arrhythmia and Electrophysiology Unit, IRCCS Fondazione Policlinico S. Matteo, Pavia, Italy.

## Supplementary material

**Consent:** The authors confirm that written consent for the submission and publication of this case, including images, has been obtained from the patient in line with the COPE guidelines.

**Funding:** None declared.
